# Prenatal dexamethasone exposure and the risk of early-onset sepsis in preterm infants

**DOI:** 10.3389/fped.2025.1673813

**Published:** 2025-10-03

**Authors:** Yulu Yang

**Affiliations:** Department of Neonatology, Women’s Hospital, Zhejiang University School of Medicine, Hangzhou, China

**Keywords:** prenatal dexamethasone exposure, early-onset sepsis, very low birth weight (VLBW), very preterm infant, antenatal corticosteroids

## Abstract

**Background:**

Robust evidence has consistently demonstrated the impact of antenatal corticosteroid (ACS) administration on reducing mortality and improving short-term neonatal outcomes in singleton preterm infants. However, its effect on neonatal sepsis, particularly early-onset sepsis (EOS), remains poorly understood and requires further investigation. This study aimed to evaluate the potential association between prenatal dexamethasone exposure (PDE) and incidence of EOS in preterm infants.

**Methods:**

This retrospective, single-center observational study included singleton preterm neonates with a gestational age less than 32 weeks or a birth weight below 1,500 g between 2022 and 2024. Participates were stratified into four groups based on PDE: no PDE, partial PDE (1–3 doses), PDE 1–7 days (complete course with delivery within 7 days of administration), and PDE ≥8 days (complete course with delivery more than 7 days after administration). The primary outcome was the incidence of EOS, while secondary outcomes encompassed other short-term neonatal complications.

**Results:**

The analysis revealed that neonates in the PDE 1–7 days group demonstrated a significantly reduced incidence of EOS compared with the no PDE group [adjusted odds ratio[aOR]: 0.299, 95% confidence interval [95%CI]: 0.122–0.731]. Furthermore, this group exhibited superior outcomes, including lower rates of respiratory distress syndrome (RDS), reduced the need for surfactant treated in RDS cases, and decreased extrauterine growth restriction (EUGR). Notably, the PDE ≥8 days group was associated with an elevated risk of EOS when compared with the PDE 1–7 days group.

**Conclusion:**

PDE, particularly when a complete course is administered 1–7 days prior to delivery, demonstrates a significant protective effect against EOS in preterm infants. Nevertheless, large-scale multicenter prospective studies are warranted to further validated these findings and to comprehensively evaluate the long-term neurodevelopmental and systemic outcomes associated with PDE administration.

## 1 Introduction

Recent advances in neonatal medicine have markedly improved the survival of preterm infants, yet very low birth weight (VLBW) and very preterm infants (VPIs) remain highly susceptible to complications like sepsis. These complications carry significant long-term implications for the child's quality of life and place a substantial burden on both families and healthcare systems.

Antenatal corticosteroids (ACS), such as dexamethasone, primarily used to mature the lungs, as a cornerstone prenatal therapy for enhancing perinatal prognosis in preterm neonates (1, 2). Their immunomodulatory effects on sepsis, however, remain a subject of debate. Prior studies have yielded inconsistent results, with some large-scale cohort studies showing no association between ACS exposure and neonatal infection (3, 4), while others suggest a potential risk of serious infections (5–7). This inconsistency largely stems from methodological heterogeneities in previous research. Specifically, prior studies often simplistically dichotomized ACS exposure as “exposed” vs. “unexposed,” failing to account for critical variables like the specific agent, the timing and completeness of the treatment, or the interval between administration and delivery. Furthermore, many studies did not differentiate between early-onset sepsis (EOS) and late-onset sepsis (LOS). These limitations in exposure definitions, treatment completeness, and study populations have obscured a clear understanding of the true immunomodulatory effects of ACS.

To address these limitations, our study employs a refined stratification of prenatal dexamethasone exposure (PDE) based on both treatment completeness and the interval between administration and delivery, allowing us to precisely examine its differential effects on early-onset sepsis. This design specifically addresses the unresolved gap in the literature by providing a more granular, time-dependent analysis of ACS immunomodulatory effects, offering crucial insights for optimizing clinical application.

## 2 Materials and methods

### 2.1 Study design and study population

This retrospective, single-center observational study analyzed data from VLBW infants and VPIs admitted in a tertiary hospital between 2022 and 2024. The study subjects comprised singleton preterm infants meeting either criterion: gestational age <32 weeks or birth weight <1,500 g. These infants were categorized into four groups based on their exposure to ACS. In accordance with relevant guidelines (8), ACS was administered to pregnant women at risk of preterm birth within one week. Our institutional protocol typically involves four doses of dexamethasone (6 mg each), administered intramuscularly at 12-h intervals. The exclusion criteria were as follows: congenital anomalies or chromosomal abnormalities, neonates who received more than one course of ACS regimen or other regimens (e.g., betamethasone), and cases with missing data or hospitalization duration less than 28 days.

We categorized neonates into four groups based on the administration of prenatal dexamethasone exposure (PDE): “No PDE” group comprised neonates born without receiving any prenatal dexamethasone; “Partial PDE” group included neonates who received 1–3 doses of dexamethasone but were born before completing the full course or within a 12-hour interval before receiving the last dose; “PDE 1–7 days” group comprised neonates who received the complete course of PDE and were born within 1–7 days of the last administration; “PDE ≥8 days” group encompassed neonates who received the complete PDE regimen and were born 8 or more days after the last administration.

### 2.2 Ethical statement

This study received approval from the Ethics Committee of Women's Hospital, School of Medicine, Zhejiang University (Approval No. IRB-20250253-R).

### 2.3 Data collection

The following data were collected from the clinical records:

Demographical variables: Gestational age at birth (based on early ultrasound or last menstrual period), birth weight, small for gestational age (SGA) (birth weight below the 10th percentile), sex, mode of delivery, 5-min Apgar score, and maternal demographics (e.g., hypertensive disorders in pregnancy (HDP), gestational diabetes mellitus (GDM), histological chorioamnionitis (hCAM), Group B Streptococcus (GBS) colonization, preterm premature rupture of membranes (PPROM) >18 h).

PDE administration details.

Neonatal complications: Defined as primary outcomes or covariates.

The definitions for each complication diagnosis were as follows: neonatal sepsis: Classified into early-onset sepsis (EOS) (<72 h after birth) and late-onset sepsis (LOS) (≥72 h after birth) (9); respiratory distress syndrome (RDS): Based on clinical manifestations and chest x-ray findings, treated RDS means the need for surfactant administration; hemodynamically significant patent ductus arteriosus (hsPDA) (10): ultrasound requires clear evidence of: (1) left-to-right shunt (or bidirectional shunt during both systole and diastole); (2) left atrium to aortic root ratio >1.3; (3) Ductus diameter >1.5 mm. And along with at least one of the following clinical signs: cardiac murmur, water-hammer pulse, tachycardia, hyperactive precordial pulsations, widened pulse pressure, or worsening respiratory status; necrotizing enterocolitis (NEC): Diagnosis based on the modified Bell's criteria (11); bronchopulmonary dysplasia (BPD): Defined according to the National Institutes of Health criteria (12); retinopathy of prematurity (ROP): Graded according to the International Classification of Retinopathy of Prematurity (ICROP) definition (13); extrauterine growth restriction (EUGR): Defined as a fall in *z*-score >1.34 (14).

### 2.4 Statistical analysis

Data were organized using Microsoft Excel and analyzed using SPSS Statistics version 27.0. Categorical variables are presented as number and percentage and comparisons were made using the chi-squared test or Fisher's exact test.

The normality of continuous variables was assessed using the Kolmogorov–Smirnov test. Normally distributed continuous variables are expressed as mean ± standard deviation (SD) and compared using one-way analysis of variance (ANOVA). Non-normally distributed measures are characterized by median (P25, P75) and analyzed with Kruskal–Wallis test with *post-hoc* testing corrected for multiple comparisons by the Bonferroni method.

Additionally, risk factors for outcomes were examined using binary logistic regression analysis with a stepwise selection method, adjusting for all relevant control variables. Adjusted odds ratios (aORs) and corresponding 95% confidence intervals (CIs) were calculated. Statistical significance was determined at a *p* value < 0.05.

## 3 Results

### 3.1 Study cohort and characteristics

A total of 755 VLBW infants and VPIs were admitted to our neonatal unit from 2022 to 2024. After applying exclusion criteria—including receipt of more than one course of dexamethasone therapy, hospitalization duration <28 days, missing data on prenatal dexamethasone exposure, and chromosomal or genetic abnormalities—145 infants were excluded. The final analysis comprised 610 neonates. Table 1 presents the characteristics of the study participants. The median gestational age and birth weight of the study population were 29.9 [28.3, 31.0] weeks and 1,261.0 ± 312.5 g, respectively. The overall rate of PDE was 95.1% (580/610), with PDE administered 1–7 days before preterm birth in 56.4% of cases.

**Table 1:** Demographics and perinatal characteristics of the groups.

Variables	Overall	No PDE	Partial PDE	PDE 1–7 days	PDE ≥8 days	Statistic	*P* value
	*n* = 610	*n* = 30	*n* = 148	*n* = 327	*n* = 105		
PDE administration							
PDE	580 (95.1)	0 (0.0)	148 (100.0)	327 (100.0)	105 (100.0)		
Gestational age at PDE (weeks)			29.8 (28.1,31.0)	29.7 (28.0,31.0)	27.9 (26.3,29,1)		
PDE-to-delivery interval (days)			0 (0,0)	2 (1,4)	10 (8,14)		
Maternal characteristics							
Advanced maternal age	167 (27.4)	10 (33.3)	39 (26.4)	85 (26.0)	33 (31.4)	1.795	0.616
Cesarean section	452 (74.1)	18 (60.0)	94 (63.5)	255 (78.0)	85 (81.0)	16.886	<0.001
HDP	200 (32.8)	7 (23.3)	24 (16.2)	123 (37.6)	46 (43.8)	28.905	<0.001
GDM	139 (22.8)	8 (26.7)	34 (23.0)	76 (23.2)	21 (20.0)	0.762	0.859
hCAM	272 (44.6)	16 (53.3)	69 (46.6)	147 (45.0)	40 (38.1)	2.986	0.394
PPROM >18 h	165 (27.0)	4 (13.3)	18 (12.2)	113 (34.6)	30 (28.6)	28.946	<0.001
GBS colonization	19 (3.1)	1 (3.3)	5 (3.4)	12 (3.7)	1 (1.0)	2.078	0.529
Neonatal characteristics							
Male	271 (44.4)	14 (46.7)	61 (41.2)	153 (46.8)	43 (41.0)	1.931	0.587
Gestational age at birth (weeks)	29.9 (28.3,31.0)	29.8 (27.3,30.7)	29.8 (28.1,31.0)	30.0 (28.4,31.3)	29.6 (28.0,30.8)	5.730	0.125
Birth weight (g)	1,261.0 ± 312.5	1,265.0 ± 369.5	1,310.7 ± 330.0	1,249.7 ± 297.5	1,225.1 ± 311.8	1.866	0.134
SGA	78 (12.8)	0 (0.0)	13 (8.8)	54 (16.5)	11 (10.5)	11.564	0.008
Apgar 5-min	10.0 (9.0,10.0)	10.0 (9.0,10.0)	10.0 (9.0,10.0)	10.0 (9.0,10.0)	10 (9.0,10.0)	11.388	0.010^a^
0–3	3 (0.5)	0 (0.0)	0 (0.0)	3 (0.9)	0 (0.0)		
4–7	27 (4.4)	4 (13.3)	4 (2.7)	12 (3.7)	7(6.7)		
8–10	580(95.1)	26(86.7)	144(97.3)	312(95.4)	98(93.3)		

Numbers are presented as *n* (%), median (P25, P75), or mean ± SD.

PDE, prenatal dexamethasone exposure; HDP, hypertensive disorders in pregnancy; GDM, gestational diabetes mellitus; hCAM, histological chorioamnionitis; SGA, small for gestational age; PPROM, preterm premature rupture of membranes; GBS, Group B Streptococcus.

a The difference between **PDE 1–7 days** and **PDE ≥8 days** groups remains statistically significant after Bonferroni correction for multiple comparisons (*p* < .05).

The four groups exhibited similar characteristics regarding advanced maternal age, GDM, hCAM, GBS colonization, infants sex, gestational age at birth and birth weight. Notably, the PDE ≥8 days group had an earlier gestational age at the time of PDE administration and at birth than the other groups. The partial PDE group and no PDE group were less likely to have undergone delivery via cesarean section. The PDE 1–7 days group comprised a higher proportion of infants with HDP and who were SGA. Patients in the no PDE and partial PDE groups were more likely to experience hCAM. Furthermore, the no PDE group was more likely to have a low Apgar score at 5 min.

### 3.2 Neonatal complications

The incidence of EOS and the other complications in each group is presented in Table 2. The PDE 1–7 days group exhibited the lowest rates of almost all complications, with the exception for LOS. PDE was associated with a statistically significant reduction in the incidence of EOS, RDS, and hsPDA, with the PDE 1–7 days group showing the lowest incidence among the four groups. Conversely, the no PDE group had the highest incidence of EOS, while the partial PDE had the highest incidence of LOS.

**Table 2:** Incidence of neonatal complications.

Complications	Overall	No PDE	Partial PDE	PDE 1–7 days	PDE ≥8 days	*X* ^2^	*P* value
	*n* = 610	*n* = 30	*n* = 148	*n* = 327	*n* = 105		
RDS	516 (84.6)	29 (96.7)	127 (85.8)	261 (79.8)	99 (94.3)	17.793	<0.001
Required ≥2 doses of surfactant	15 (2.5)	2 (6.7)	4 (2.7)	7 (2.1)	2 (1.9)	2.726	0.408
Treated RDS	303 (49.7)	18 (60.0)	78 (52.7)	142 (43.4)	65 (61.9)	13.214	0.004
hsPDA	137 (22.5)	8 (26.7)	44 (29.7)	60 (18.3)	25 (23.8)	8.080	0.044
NEC	31 (5.1)	1 (3.3)	12 (8.1)	13 (4.0)	5 (4.8)	3.484	0.290
Need for inotropes or fluid Resuscitation	131 (21.5)	10 (33.3)	33 (22.3)	62 (19.0)	26 (24.8)	4.460	0.217
EOS (<72 h)	109 (17.9)	9 (30.0)	32 (21.6)	40 (12.2)	28 (26.7)	17.045	<0.001
LOS (≥72 h)	137 (22.5)	4 (13.3)	40 (27.0)	68 (20.8)	25 (23.8)	3.838	0.280
BPD	271 (44.4)	16 (53.3)	64 (43.2)	138 (42.2)	53 (50.5)	3.260	0.353
ROP	233 (38.2)	14 (46.7)	60 (40.5)	119 (36.4)	40 (38.1)	1.708	0.641
EUGR	115 (18.9)	10(33.3)	33(22.3)	51(15.6)	21(20.0)	7.617	0.053

PDE, prenatal dexamethasone exposure; RDS, respiratory distress syndrome; hsPDA, hemodynamically significant patent ductus arteriosus; NEC, necrotizing enterocolitis; EOS, early-onset sepsis; LOS, late-onset sepsis; BPD, bronchopulmonary dysplasia; ROP retinopathy of prematurity; EUGR, extrauterine growth restriction.

Variables are given as number and percentage.

In the adjusted logistic regression model (Table 3), the PDE 1–7 days group showed a significantly decreased aOR for EOS [aOR: 0.299 (95% CI: 0.122, 0.731)] compared with the no PDE group. The PDE 1–7 days group also demonstrated significantly decreased aOR for RDS [aOR: 0.217 (95% CI: 0.028, 0.694)], treated RDS [aOR: 0.679 (95% CI: 0.346, 0.958)] and EUGR [aOR: 0.423 (0.182, 0.982)] compared with the no PDE group.

**Table 3:** Logistic regression analysis for preterm morbidities.

Complications	aOR [95% CI]^a^		
	Partial PDE	Complete PDE	
		PDE 1–7 days	PDE ≥8 days
RDS	0.312 (0.039, 2.484)	0.217 (0.028, 0.694)	0.684 (0.075, 4.207)
Treated RDS	0.973 (0.421, 2.253)	0.679 (0.346, 0.958)	1.359 (0.563, 3.284)
hsPDA	1.533 (0.617, 3.811)	0.802 (0.327, 1.967)	0.968 (0.369, 2.537)
EOS (<72 h)	0.717 (0.292, 1.763)	0.299 (0.122, 0.731)	0.820 (0.323, 2.078)
EUGR	0.648 (0.270, 1.552)	0.423 (0.182, 0.982)	0.563 (0.223, 1.418)

aOR, adjusted odds ratio; CI, confidence interval; PDE, prenatal dexamethasone exposure; RDS, respiratory distress syndrome; hsPDA, hemodynamically significant patent ductus arteriosus; EOS, early-onset sepsis; EUGR, extrauterine growth restriction.

a Model adjusted for type of delivery, HDP, SGA, 5-min Apgar score, PPROM >18 h; no PDE group as the reference group.

## 4 Discussion

With advancements in medical science, the survival rate of preterm infants has gradually increased. However, this progress has also been accompanied by a rise in the incidence of complications among preterm infants. VLBW infants and VPIs face significantly higher risks of complications and mortality (15). EOS remains a serious and often fatal illness among preterm infants, with a contemporary incidence of approximately 13–18 cases per 1,000 preterm births at gestational age under 29 weeks (16, 17). With an associated all-cause mortality rate as high as 35%, it is one of the leading causes of neonatal morbidity and mortality (18). A cross-sectional survey of discharge outcomes for VLBW infants in 25 tertiary-level NICU wards in China in 2018 (19) revealed that among the disease incidence rates in VLBW infants, sepsis ranks first among complications such as BPD, ≥Grade III intraventricular hemorrhage or periventricular leukomalacia (PVL), ≥Stage II NEC, and ROP. This undoubtedly poses a severe threat to the survival quality of preterm infants and imposes a significant economic burden on society.

Glucocorticoids are a crucial class of regulatory factors in the body, playing a vital role in regulating development, growth, metabolism, and immune function. They possess various biological functions, including anti-inflammatory, antitoxic, antiallergic, suppression of nonspecific immune responses, and anti-shock effects (20). As early as the 1970s, researchers confirmed that prenatal glucocorticoid therapy could promote fetal lung maturation and significantly reduce the incidence and mortality of neonatal respiratory distress syndrome (21). The 2018 “Expert Consensus on Early Prevention and Treatment of Neonatal Respiratory Distress Syndrome (NRDS)” (22) recommends that for pregnancies with a risk of preterm delivery within 7 days, glucocorticoids should be administered prenatally to promote fetal lung maturity based on clinical circumstances. The optimal timing for administration is between 24 h and 1 week before delivery. While prenatal glucocorticoid use has gained widespread acceptance, its potential impacts on fetuses and preterm infants remain controversial. Moreover, evaluating the benefits and risks of prenatal glucocorticoid application is influenced by numerous confounding factors, and preterm infants inherently face multiple organ damage risks.

Multiple studies have investigated whether prenatal glucocorticoid therapy increases the risk of neonatal infection, but current reports remain inconsistent. A previous nationwide cohort study found that both preterm and term infants whose mothers received a single course of corticosteroid treatment during pregnancy had a significantly increased risk of severe infection within 12 months after birth (23). Similarly, Lee et al. analyzed data from 176,681 mother-infant pairs across Taiwan between 2010 and 2019 and found that steroid usage was an independent risk factor for EOS in preterm infants (24). One study (25) (2,549 newborns) found increased odds of neonatal sepsis at ACS 2–7 days (OR: 1.39, 95% CI: 1.07–1.81) and ACS >7 days (OR: 1.32, 95% CI: 1.12–1.56). Conversely, the majority of researchers believe that prenatal use of glucocorticoids does not significantly increase the risk of infection in preterm infants. Harding et al.'s meta-analysis (26) showed that prenatal glucocorticoid use in patients with PPROM had almost no impact on the risk of neonatal or maternal infection, which is consistent with the findings of Elimian et al. (4). Additionally, neonatal sepsis did not increase after a single course of ACS; however, multiple courses of treatment may increase the risk of chorioamnionitis and early-onset neonatal sepsis (27, 28). A 2020 domestic retrospective study showed that prenatal application of corticosteroids did not affect the incidence of sepsis or intrauterine infectious pneumonia in preterm infants (29). However, this study did not further stratify maternal premature rupture of membranes duration, thus limiting the generalizability of its conclusions. Fuma et al. (3) analyzed data from singletons born prematurely at two tertiary medical centers in Japan over several years and they found that partial ACS administration showed no significant effect on outcomes compared with the no ACS group but showed a higher trend in neonatal sepsis. While some studies have reported contradictory results, others have revealed a beneficial effect. Ertekin recently proposed that administering glucocorticoids within one week before birth significantly reduces the incidence of EOS in preterm infants born between 24 and 30 weeks' gestation (aOR: 0.18, 95% CI: 0.067–0.52, *p* = 0.001) (30). However, given the small number of subjects, the power of this study is limited, making it less compelling.

The discrepancy among these findings may stem from differences in study design. First, this study stratified PDE into four groups based on both dosage (partial vs. complete course) and time interval (1–7 days vs. ≥8 days), whereas most previous studies only compared “exposed vs. unexposed” without distinguishing dosage completeness or timing. For example, Wong et al. (25) reported increased sepsis risk in ACS-exposed infants but did not account for the interval between administration and delivery, which may mask the protective effect of the 1–7 day window. Second, this study focused on singleton preterm infants with strict inclusion criteria (<32 weeks' gestation or birth weight <1,500 g), reducing confounding from term infants or multiple pregnancies—factors that may have influenced results in population-based studies like Liang et al (7). Notably, our study demonstrates that PDE, particularly a complete course administered 1–7 days before birth, is significantly associated with a reduced risk of EOS in preterm infants (aOR = 0.299, 95% CI: 0.122–0.731). This finding is consistent with Ertekin et al. (30), who observed that ACS within 1 week lowers severe complications in preterm infants, but our study further quantified this protective effect through more refined subgroup analysis and confirmed the clinical significance of the precise “1–7 day” therapeutic window. In contrast, Fuma et al. (3) found no significant benefit of partial ACS, which aligns with our observation that the partial PDE group had higher EOS incidence than the PDE 1–7 days group, highlighting the importance of completing the full course.

Concurrently, the PDE 1–7 days group showed lower incidences of RDS and EUGR, indicating that PDE within this time window may exert comprehensive protective effects on preterm infants' short-term outcomes. The time-dependent effect of PDE (1–7 days vs. ≥8 days) suggests a critical “therapeutic window” for dexamethasone. This may be attributed to the temporal dynamics of glucocorticoid action: dexamethasone promotes fetal lung maturation and modulates immune responses within 24 h to 7 days after administration, enhancing the fetus's ability to resist perinatal infections and reducing the risk of EOS triggered by immature immune function. In contrast, when the interval exceeds 7 days, the drug's biological activity may diminish, and prolonged exposure might weaken the immune regulatory effect, which aligns with the higher EOS incidence in the PDE ≥8 group compared to the PDE 1–7 days group. Previous animal experimental studies have shown that ACS suppress endotoxin-induced inflammation within one day of treatment. However, paradoxically, the inflammatory response is amplified at five and fifteen days post-treatment (31). The incidence rates of RDS and treated RDS in the various groups of this study also suggest this phenomenon. The RDS incidence rate was lowest in the PDE 1–7 days group, lower in the PDE ≥8 days group compared to the no PDE group, but higher than in the partial PDE group. This finding aligns with a previously published review (8). We similarly observed that the incidence of BPD was higher in the PDE ≥8 days group in this study. This suggests that while PDE may prevent RDS, prolonged exposure may be detrimental to subsequent lung development and could even be associated with an increased risk of BPD, consistent with findings reported by Carlo et al. (32, 33). Previous literature on the effects of ACS on the immune system appears to explain this phenomenon. One study examined human leukocyte DR antigen expression in 56 extremely low birth weight infants with gestational ages <32 weeks. It found that the degree of immune suppression induced by ACS is related to the time interval between drug administration and delivery. Administration of ACS within 24 h before delivery increases the likelihood of early preterm infants having a human leukocyte DR antigen expression rate <60%, placing them in an immunosuppressed state. As the interval between drug administration and delivery increases, the risk of immunosuppression decreases (34). A retrospective cohort study analyzed levels of inflammatory factors such as C-reactive protein in peripheral blood from preterm infants with gestational ages <28 weeks. It demonstrated that ACS exerts anti-inflammatory effects in preterm infants during the first week after birth, gradually weakening by day 28, suggesting that ACS can regulate the inflammatory response in the short term (35).

Although univariate analysis showed no significant association between PDE and EUGR (*p* = 0.053）, multiple multicenter retrospective studies have confirmed that ACS provides significant protection against EUGR in preterm infants and is associated with faster weight gain in extremely low birth weight infants (36, 37). Therefore, we included it in multivariate analysis. To our surprise, we found that PDE 1–7 significantly reduced the incidence of EUGR (aOR = 0.423, 95% CI: 0.182–0.982). Based on previous research, this phenomenon may be explained by ACS promoting gastrointestinal maturity (e.g., increased gastrin secretion), improving feeding tolerance, and indirectly supporting growth by reducing severe respiratory diseases (38, 39).

This study has several limitations. Firstly, maternal antibiotic usage-a key factor in neonatal infection risk-was not recorded, potentially biasing the EOS analysis, especially if PDE groups had differential antibiotic exposure. In this study, the PDE 1–7 group had a higher incidence of PPROM >18 h, which, according to clinical guidelines, would typically lead to more extensive or earlier intrapartum antibiotic exposure. Although detailed data on antibiotic use were not collected in this study, this factor likely contributed in part to the lower EOS rate observed in this group. Secondly, the small sample size in the no PDE group, may reduce the statistical power for between-group comparisons. Additionally, excluding infants exposed to multiple courses of prenatal dexamethasone, though methodologically necessary to control for confounding and ensure a homogeneous exposure group, may limit generalizability. Our findings thus apply mainly to single-course exposures and not to multiple-course recipients, who often have more severe maternal conditions and potentially higher infection risk. Third, data collection occurred during the COVID-19 pandemic, which introduced unmeasured confounders. Maternal SARS-CoV-2 infection-linked to preterm delivery (40) was not systematically documented, precluding adjustment. Pandemic-related infection control measures (e.g., visitor restrictions, improved hygiene) may also have affected neonatal infection rates independently of pharmacological interventions (41, 42). Finally, in Table 2, although no significant difference in LOS incidence was found between groups (*p* = 0.280), this result should be interpreted cautiously due to the lack of adjustment for major nosocomial risk factors such as duration of mechanical ventilation, central venous catheters, and parenteral nutrition. These omissions may obscure the true association between prenatal exposure and outcome.

## 5 Conclusions

In summary, our findings demonstrate that a complete course of antenatal dexamethasone administered 1–7 days before birth is associated with reduced incidence of EOS, RDS, surfactant administration, and EUGR in very low birth weight and very preterm infants. These results reinforce the importance of deliberate, well-timed antenatal corticosteroid use in women at risk of preterm delivery.

Clinically, our data support current guidelines emphasizing timely ACS administration while highlighting a potential therapeutic window that may optimize neonatal outcomes. However, these conclusions should be interpreted considering possible residual confounding—such as unaccounted maternal antibiotic exposure or other unmeasured clinical variables—as well as limitations in small simple size and potential risks of overfitting given the number of comparisons. Future multi-center prospective studies with larger, more diverse cohorts are needed to validate the 1–7 day treatment window, adjust more thoroughly for confounders, and evaluate long-term neurodevelopmental and health outcomes.

## Data Availability

The raw data supporting the conclusions of this article will be made available by the authors, without undue reservation.
